# Hunting for hydrogen: random structure searching and prediction of NMR parameters of hydrous wadsleyite†
†Electronic supplementary information (ESI) available: Further information on the structures generated by AIRSS, alternative structural models, supercell calculations, total enthalpies of all computed structures and further information on ^1^H/^2^H NMR parameters. Example input and all raw output files from AIRSS and CASTEP NMR calculations are also included. See DOI: 10.1039/c6cp01529h



**DOI:** 10.1039/c6cp01529h

**Published:** 2016-03-29

**Authors:** Robert F. Moran, David McKay, Chris J. Pickard, Andrew J. Berry, John M. Griffin, Sharon E. Ashbrook

**Affiliations:** a School of Chemistry , EaStCHEM and Centre of Magnetic Resonance , University of St Andrews , St Andrews KY16 9ST , UK . Email: sema@st-andrews.ac.uk; b Department of Materials Science & Metallurgy , University of Cambridge , 27 Charles Babbage Road , Cambridge CB3 0FS , UK; c Research School of Earth Sciences , Australian National University , Canberra , ACT 2601 , Australia; d Department of Chemistry , Lancaster University , Lancaster LA1 4YB , UK

## Abstract

The structural chemistry of materials containing low levels of nonstoichiometric hydrogen is difficult to determine, and producing structural models is challenging where hydrogen has no fixed crystallographic site. Here we demonstrate a computational approach employing *ab initio* random structure searching (AIRSS) to generate a series of candidate structures for hydrous wadsleyite (β-Mg_2_SiO_4_ with 1.6 wt% H_2_O), a high-pressure mineral proposed as a repository for water in the Earth's transition zone. Aligning with previous experimental work, we solely consider models with Mg3 (over Mg1, Mg2 or Si) vacancies. We adapt the AIRSS method by starting with anhydrous wadsleyite, removing a single Mg^2+^ and randomly placing two H^+^ in a unit cell model, generating 819 candidate structures. 103 geometries were then subjected to more accurate optimisation under periodic DFT. Using this approach, we find the most favourable hydration mechanism involves protonation of two O1 sites around the Mg3 vacancy. The formation of silanol groups on O3 or O4 sites (with loss of stable O1–H hydroxyls) coincides with an increase in total enthalpy. Importantly, the approach we employ allows observables such as NMR parameters to be computed for each structure. We consider hydrous wadsleyite (∼1.6 wt%) to be dominated by protonated O1 sites, with O3/O4–H silanol groups present as defects, a model that maps well onto experimental studies at higher levels of hydration (J. M. Griffin *et al.*, *Chem. Sci.*, 2013, **4**, 1523). The AIRSS approach adopted herein provides the crucial link between atomic-scale structure and experimental studies.

## Abstract 2

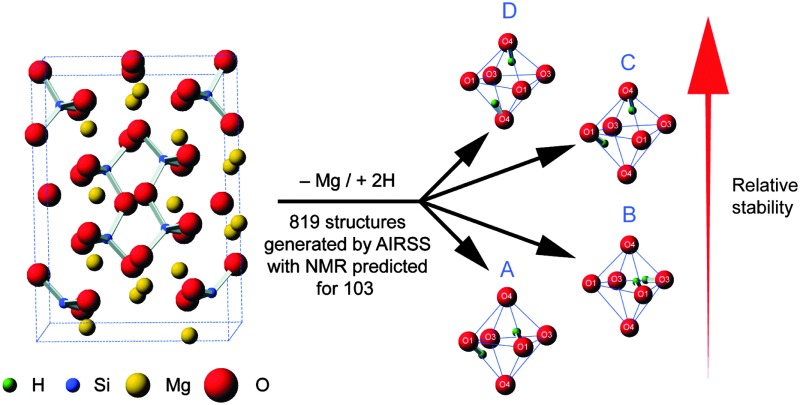
 *Ab initio* random structure searching is employed to generate candidate structures of hydrous wadsleyite, predicting NMR parameters for experimental comparison.

## Introduction

While disorder in solids often leads to interesting physical and chemical properties, its presence can make it very challenging to characterise (and even to describe) “the structure”. The average structural models produced by some experimental approaches can provide insight, but may raise as many questions as they are able to answer. For example, if a site is stated to have a fractional occupancy of 0.5 in a structural model, this may mean it is occupied in half of the unit cells, or it is occupied for half of the time, *i.e.*, static or dynamic disorder. In principle, the dependence of NMR spectroscopy on the local structure makes it an ideal probe of disorder in the solid state.^1^ However, the presence of anisotropic broadening (averaged by rapid molecular tumbling in solution-state NMR) restricts resolution and the extraction of detailed information. A number of methods exist to remedy this – including magic-angle spinning (MAS), high-power decoupling and, for nuclei with *I* > 1/2, experiments such as multiple-quantum MAS, which are designed to remove the quadrupolar interaction these species experience.^1,2^ Even when high-resolution spectra are obtained, the spectral lineshapes for disordered materials are often complicated, with many overlapped or unresolved components, owing to the distribution of NMR parameters that result from the variations in local environment. Such spectra still present a considerable challenge to interpret and assign.^1,2^

In recent years, there has been growing interest in the use of theoretical calculations to provide a prediction of the NMR parameters and to validate structural models in conjunction with experimental measurements.^3–5^ In particular, the GIPAW^6^ approach, implemented in planewave density functional theory (DFT) codes such as CASTEP,^7–9^ has been applied to a range of materials, including organic pharmaceuticals, microporous solids, energy materials and supramolecular assemblies, with excellent agreement between experiment and calculation for many different nuclear species.^3–5^ The majority of studies have considered well-ordered crystalline solids, with initial structural models provided by prior diffraction-based experiments or previous computational work. Optimisation of the geometry can then be used to ensure that the lowest energy configuration of atoms is used. Generating an initial model is, however, more complex when considering disordered solids. The average structures that result from diffraction – often with fractional or partial site occupancies – cannot be used directly, and multiple structural models can have similar energies. Possible solutions to this problem include the simple substitution of one or more atoms into a known but ordered structure, thereby providing information on the changes in NMR parameters that are expected upon such substitution, or the generation of a set of similar structures with atoms placed on different sites, and the predicted NMR parameters from each then summed. Although these approaches have seen much success,^3–5,10–16^ they quickly become unfeasible as the amount and types of disorder increase resulting in an increasingly large number of possible structures. Furthermore, for some disordered systems atoms may not lie at well-defined sites, leading to a potentially infinite range of possible structures. Choices have to then be made about how possible models are generated, whether all possibilities are considered equally or whether only those that appear most “chemically reasonable” or have lower energy are selected. Any constraint placed on the structures considered may lead to bias in the results, or in the omission of potentially important motifs.

Recent advances in computing power, and improvements in the accuracy with which energies can be calculated at reasonable costs, have resulted in structure prediction, *i.e.*, searching for stable structures of materials, becoming a rapidly-expanding field.^17^ At a fundamental level this provides a method for determining the most stable structure, or global minimum, but these approaches can also provide information on local energy minima and potential structural variants (of considerable interest for disordered solids). Computational searching can be easier and cheaper than experiments, and can also augment incomplete or ambiguous experimental data. Searches can also be performed under conditions that are difficult to reproduce experimentally. The *ab initio* random structure searching (AIRSS)^18–21^ approach utilizes first-principles DFT in a simple strategy for generating essentially random initial configurations. The simplest searches involve a randomly selected unit cell with the positions of the atoms then chosen randomly. First-principles methods are then used to relax each structure to an enthalpy minimum. The process is then repeated many hundreds or thousands of times. As the complexity of the structure increases constraints will need to be employed, perhaps using known initial cell sizes, restricting species to have a particular bonding environment within the final structure, or adding prearranged molecules or groups of atoms.^19^

**Fig. 1:**
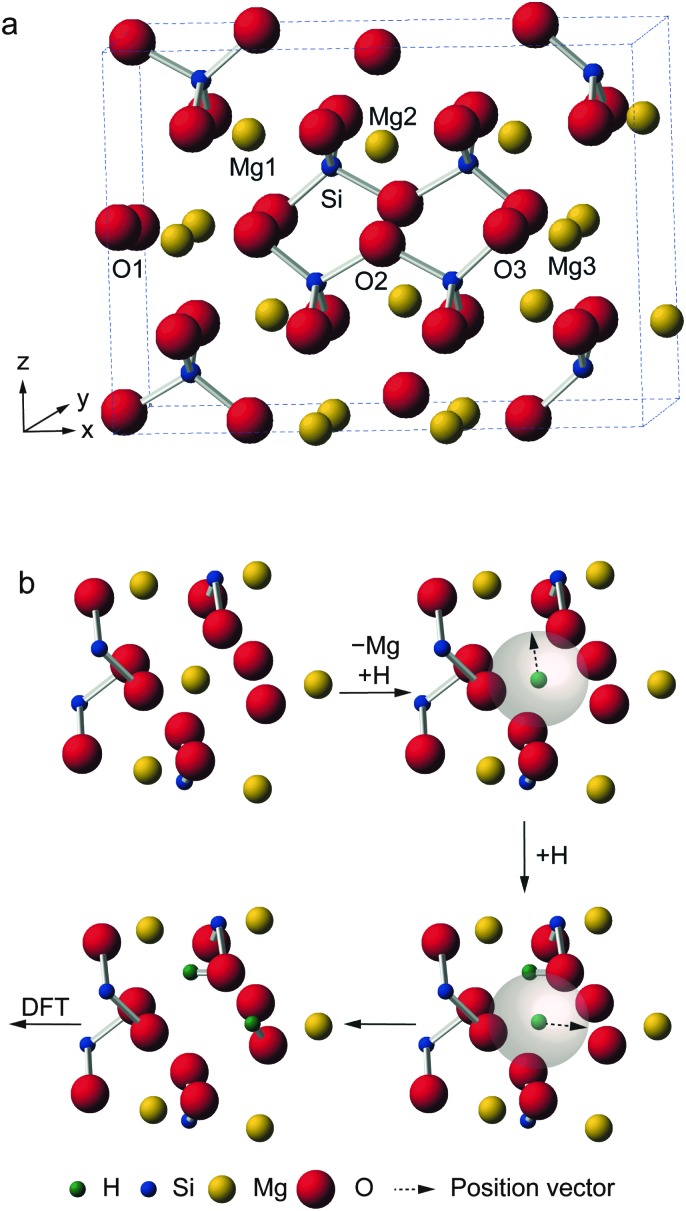
(a) Structure of anhydrous Fe-free wadsleyite, showing the crystallographic sites. (b) Schematic showing a globally-constrained, local AIRSS process, replacing one Mg3 cation in the anhydrous wadsleyite structure (7 Å cluster shown) with two H^+^ cations, which are placed randomly in a 3.0 Å sphere, before optimization using DFT.

Here, we utilize AIRSS to help investigate a particularly challenging experimental problem – the structural characterisation of hydrous wadsleyite. Fe free wadsleyite (β-Mg_2_SiO_4_, Fig. 1a) is the Mg end-member of wadsleyite (β-(Mg,Fe)_2_SiO_4_), a high-pressure mineral that is believed to be the principal component of the Earth's transition zone, between depths of ∼410 and 530 km.^22–25^ Over the last few decades, it has been recognised that the nominally anhydrous minerals (NAMs) that make up the Earth's mantle can hold significant quantities of hydrogen as hydroxyl units, typically referred to as “water”, as defects in the structure, resulting in significant effects on the physical and chemical properties of the Earth.^26,27^

Wadsleyite has received intense interest as a potential water reservoir, as it can incorporate up to 3.3 wt% H_2_O, a much greater amount than the other mantle minerals.^22–25^ The substitution of hydrogen into wadsleyite is charge balanced by the removal of a Mg^2+^ cation, but there is no fixed crystallographic site upon which the hydrogen is expected to be located. A variety of work has appeared in the literature attempting to address this problem, using both experimental approaches including XRD, neutron diffraction, FTIR and solid-state NMR, and also computation.^28–42^ The high pressures required for synthesis (14–15 GPa) present technical challenges and also limit the amounts of sample that can be produced. While there is some broad agreement on probable structural models, for example, that vacancies are located on Mg3 sites,^28,36,37,40^ there remain a number of different defect types proposed, some debate over which oxygen species are protonated and disagreement over the exact location of H atoms. It should be noted that in some previous computational studies potential docking sites were considered without explicit inclusion of cation vacancies.^29,30^ Although our recent NMR study^40^ did attempt to relate the experimental NMR parameters to those predicted by DFT, only a small number of initial structural models were used and their selection was (by necessity) biased by previous literature suggestions. In this work, we use AIRSS to provide a range of possible structural models (and information on their relative energies), independently of any prior experimental measurements. We have adapted the original AIRSS approach by starting from an initial anhydrous wadsleyite structure, removing a single Mg^2+^ cation and randomly placing two H^+^ within 3.0 Å of the vacancy (see Fig. 1b). In addition to providing possible structural models, as AIRSS also uses the CASTEP code, the NMR parameters for the proposed models can be directly calculated and compared to experiment, aiding spectral assignment and interpretation for this challenging material.

## Computational methodology

Structural models of hydrous wadsleyite were generated using AIRSS.^18,19^ Starting with the structure of anhydrous wadsleyite (*Imma* with 8 Mg_2_SiO_4_ formula units per unit cell)^43^ a random structure search was performed, where the positions of all Mg, Si and O atoms and the unit cell dimensions were predetermined, while H atoms were positioned randomly using AIRSS. The substitution of 1 × Mg by 2 × H atoms (corresponding to a hydration level of 1.6 wt%) was achieved by removing a single Mg3 cation and sequentially placing each H atom on the now vacant site and randomly moving it to a position within a sphere of defined radius (chosen to be 3.0 Å), as shown in Fig. 1b. A series of 819 hydrous wadsleyite structures, each with a single Mg3/2H substitution, was produced. Each structure generated through this procedure was then used as the repeating unit in periodic DFT calculations using the CASTEP code (version 7.0).^7^ All calculations used the PBE exchange–correlation functional^44^ of with ultrasoft pseudopotentials^8,45^ and planewave basis sets. This methodology has been shown to be of sufficient accuracy in previous studies using the AIRSS approach.^19^ For initial geometry optimisations, Brillouin space was sampled using *k*-points generated on a Monkhorst–Pack grid with a default spacing of 0.1 × 2π Å^–1^, and a default planewave cut-off energy, *E*_cut_, of 25 Ry was used. All internal atomic coordinates were allowed to vary while lattice parameters were fixed. While at this stage, low computational expense was preferred over accuracy, previous studies have shown that poor sampling of the Brillouin zone (*i.e.*, a low number of *k*-points) can lead to artifacts whereby unphysical structures are retained.^19^ For more detail on the generation of structures using AIRSS see ESI.†

Initial optimised structures (819 examples) were ranked according to relative enthalpy (Δ*H*) with the lowest set to Δ*H* = 0.0 eV. This was then used to select structures for further study. These included the 25 most stable structures, then one structure for each of a series of increments (*δ*Δ*H*): *δ*Δ*H* = 0.002 eV up to Δ*H* = 0.5 eV; *δ*Δ*H* = 0.005 eV for 0.5 < Δ*H* < 0.6 eV; *δ*Δ*H* = 0.05 eV for 0.6 < Δ*H* < 1.0 and *δ*Δ*H* = 0.1 eV for each structure with a relative enthalpy above 1.0 eV. This selection process ensured a large number of relatively stable structures were sampled. In addition, a number of less stable structures are included to allow for comparisons across the full enthalpy range. These structures were then optimised using better converged values of *E*_cut_ (60 Ry) and *k*-point grid spacing (0.04 × 2π Å^–1^, resulting in 30 *k*-points). All internal atomic coordinates and the lattice parameters were allowed to vary. This produced a set of 103 “fully-optimised” structures. NMR parameters were then computed for these structures using the GIPAW^6^ algorithm within CASTEP at a level of theory consistent with the full optimisations. Post processing of computed NMR data was conducted using Python scripts extending the CCP-NC MagresPython module.^46^ Calculations were performed using a cluster at the University of St Andrews, consisting of 201 AMD Opteron processing nodes, each containing twelve cores, partly connected by Infinipath high-speed interconnects. Calculation wallclock times ranged from 8 to 48 h using 72 cores.

Calculations of the NMR parameters produce the absolute shielding tensor (**σ**) and the quadrupolar tensor (**V**). Diagonalisation yields the three principal components, *σ*_11_, *σ*_22_ and *σ*_33_ and *V*_*XX*_, *V*_*YY*_ and *V*_*ZZ*_. The isotropic chemical shift *δ*_iso_, is given by (*σ*_ref_ – *σ*_iso_), where *σ*_iso_ is (⅓) Tr(**σ**) and *σ*_ref_ is a reference shielding. Reference shielding values of 31.0, 320.1, 560.1 and 249.7 ppm were used for ^1^H, ^29^Si, ^25^Mg and ^17^O, respectively, determined by comparison of experimental and computational results for Mg(OH)_2_ (^1^H), MgO (^25^Mg) and anhydrous wadsleyite (^29^Si and ^17^O). From the principal components of **σ** (*σ*_11_ ≤ *σ*_22_ ≤ *σ*_33_), the magnitude (or span), *Ω* = *σ*_33_ – *σ*_11_, and the skew, *κ* = 3(*σ*_iso_ – *σ*_22_)/*Ω*, can also be determined. The quadrupolar coupling constant, *C*_*Q*_ = *eQV*_*ZZ*_/*h* and the asymmetry parameter, *η*_*Q*_ = (*V*_*XX*_ – *V*_*YY*_)/*V*_*ZZ*_, are obtained directly from the principal components of **V**. *Q* is the nuclear quadrupole moment (for which values of 199.4 and –25.6 mb were used for ^25^Mg and ^17^O, respectively).

## Results and discussion

### 1. Structure searching

**Fig. 2:**
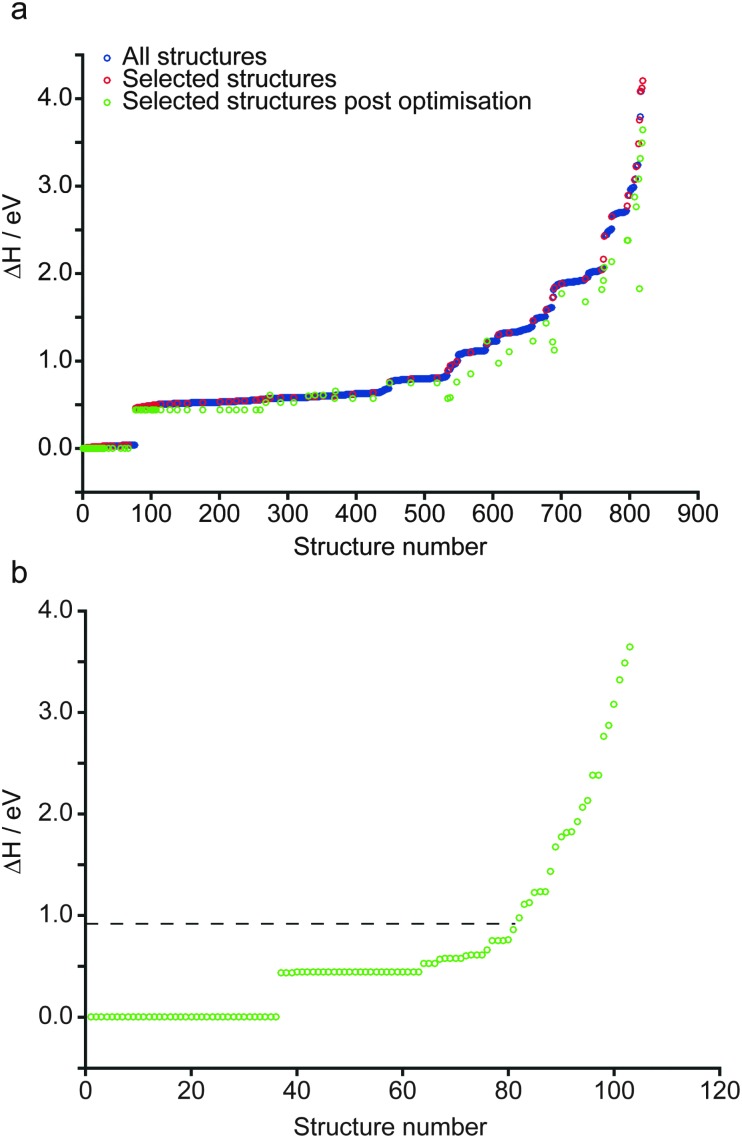
(a) Plot showing the relative enthalpy of all 819 (blue) and 103 systematically selected (red) AIRSS-generated structures of hydrous wadsleyite with an initial Mg3 vacancy. The relative enthalpy of the 103 structures, post DFT geometry optimisation is also shown (green). (b) Plot showing the 103 selected structures re-normalised according to the enthalpy after optimisation. The dotted line represents the relative enthalpy of the structural model derived from that proposed by Smyth.^24^

The structure of anhydrous wadsleyite, shown in Fig. 1a, contains Si_2_O_7_^4–^ (*i.e.*, pyrosilicate) groups. There are four crystallographically-distinct oxygen species within the structure – O1 (an unusual species coordinated by five Mg^2+^ cations), O2 a bridging (*i.e.*, Si–O–Si) oxygen and two non-bridging oxygens, O3 and O4. Of the three different Mg^2+^ cations that could possibly be removed upon hydration, common consensus (from computational work and diffraction experiments) is that hydrous wadsleyite contains Mg3 vacancies,^28,36,37,40^ though Mg2 vacancies have also been predicted.^20^ In this work we have generated structures assuming a single Mg3 vacancy per unit cell (corresponding to 1.6 wt% hydration), while structures with Mg1 and Mg2 vacancies will provide a route for future investigations. In the present study, a series of 819 structures with Mg3 vacancies were generated (see Computational methodology and Fig. 1b), where two H atoms were randomly positioned within a 3.0 Å radius of the vacancy. This radius was chosen to enable a large range of structures to be generated, while mitigating the formation of highly unstable structures, by limiting the charge separation. As a result, protonation of oxygen sites (2 × O1, 2 × O3 and 2 × O4) on the octahedron around the Mg3 vacancy was favoured. Eleven structures were generated that contained a single proton outside this octahedron (1 × H–O2, 4 × H–O3 and 6 × H–O4), and three had both protons outside the octahedron (bonded to O3 or O4 sites). These were found to be relatively unstable; all fourteen structures had Δ*H* from *ca.* 1.0 to 3.6 eV. Moreover, the two most unstable structures were those with two protons bonded to oxygen atoms outside the octahedron. Using this approach it was found that just one of the 819 structures contained an O2 hydroxyl (see ESI,† Fig. S2.1). Further, this structure was associated with a significant destabilisation (Δ*H* = 1.9 eV) and indeed no evidence of O2 protonation has been reported in the experimental literature.^40^ The enthalpies of all structures generated, sorted by Δ*H*, are plotted in Fig. 2a. Structures selected for further analysis are shown in red; Δ*H* values of fully-optimised geometries (re-normalised to the lowest enthalpy fully-optimised geometry) are shown in green. For the structures with the lowest enthalpy, the Δ*H* values remain similar after optimisation, with only small decreases due to the constraints applied to the unit cell in the initial calculations being released. More variation is observed for structures with the higher initial enthalpies, with the largest changes corresponding to structures where the substituted ^1^H moved significantly during the second optimisation step. Fig. 2b shows re-sorted Δ*H* values corresponding to the final set of 103 fully-optimised structures.

**Fig. 3:**
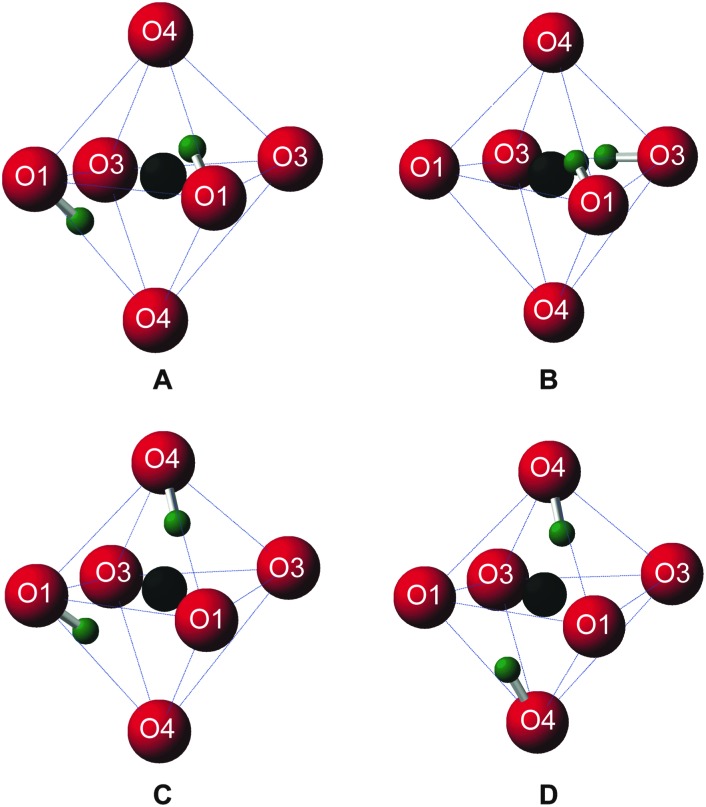
Structural motifs extracted from four representative structures generated by AIRSS, centered on the Mg3 vacancy, showing: **A**, two O1 hydroxyls; **B**, one O1 and one O3 hydroxyl; **C**, one O1 and one O4 hydroxyl and **D**, two O4 hydroxyls. H, O and vacancy position shown in green, red and black, respectively.

Upon inspection, the structures with the lowest enthalpy (representative structure, **A** (Δ*H* = 0.0 eV) shown in Fig. 3) correspond to those where only the O1 site is protonated. These structures contain hydroxyl groups staggered with respect to each other (dihedral angle, *d*(HOOH) = 104° in **A**), lying along O1–H···O4 vectors, hydrogen bonded to O4, in good agreement with previous studies.^32–34^ The next plateau of points shown in Fig. 2b at Δ*H* ≈ 0.5 eV, corresponds to structures containing adjacent protonated O1 and O3 sites, *e.g.*, **B**, in Fig. 3. The protonation of a pyrosilicate oxygen site (O3) rather than an O1 site appears to be associated with a sizeable decrease in stability, perhaps unsurprisingly since, as a result, this structural motif contains a formally “underbonded” O1. Hydroxyl bond vectors are again orientated along the edges of the vacant octahedron, here O1–H···O4 and O3–H···O3 interactions are found with *d*(HOOH) = 78.4°. The protonation of an O4, rather than an O3 site, giving motif **C**, is associated with a further enthalpy increase of ∼0.1 eV. Here, O1–H···O4 and O4–H···O1 hydrogen bonding interactions are seen (HOOH dihedral angle of 40° in **C**), possibly suggesting a relationship between **A** and **C** through hopping of a proton across the O1–H···O4 hydrogen bond. The trend in relative enthalpy with respect to protonation site (O1–H < O3–H < O4–H) is associated with the nature of the newly-formed hydroxyl, with an increase in bond length, from 0.99 to 1.03 Å, and a decrease in Mulliken bond population, from 0.66 to 0.60, across the series (see Table 1). The latter indicates a decrease in bond covalency. While the range in relative enthalpy seen here is not insignificant (*ca.* 0.6 eV), this is thought to be due to the explicitly periodic nature of the model used, whereby protons in non-optimal environments (*i.e.*, silanol groups) and optimal positions (*i.e.*, O1-bound) are necessarily present in a 1 : 1 ratio, resulting in a large destabilisation.

**Table 1:** O–H bond distances and Mulliken bond populations, showing the effect of O site on the nature of the newly-formed hydroxyl group. Structures **A**, **B** and **C** refer to the models shown in Fig. 3

Structure	O–H distance/Å (O site)	O–H bond population
**A**	0.99 (O1)	0.66
	0.99 (O1)	0.66
**B**	0.99 (O1)	0.66
	1.01 (O3)	0.62
**C**	0.99 (O1)	0.66
	1.03 (O4)	0.60

Test calculations were conducted considering larger periodic repeat units (derived from 2 × 2 × 1 supercells with 32 Mg_2_SiO_4_ formula units, therefore allowing 4 × Mg3 vacancies and 8 × H atoms; see ESI†), through which a reduced silanol to O1–H ratio of 1 : 7 was modelled. These suggest silanol groups seen experimentally may be present as “defects” within a structure dominated by O1–H hydroxyls, where silanol formation, though coming at a high enthalpic penalty, becomes feasible through the increase in entropy as the silanol to O1–H ratio decreases.

A periodic system entirely devoid of O1–H hydroxyls is considered to be unlikely. Such models, *e.g.*, **D** in Fig. 3, exhibit enthalpies of at least 0.8 eV above O1–H hydroxyl-dominated congeners.

A previous study by Smyth suggested a structure for fully hydrous (3.3 wt%) wadsleyite, where 2 × Mg2 cations were substituted for 4 × H, with all protonation at the O1 sites.^24^ However, due to the ionic point charge model and symmetry constraints adopted in that study, O1–H hydroxyls were found to align with the *z*-axis (as defined in the present work), rather than forming hydrogen-bonding interactions.^24^ A model based on the Smyth structure, modified to represent the 1.6 wt% hydration level considered here, was generated and fully optimised (see ESI† for details). Its relative enthalpy (Δ*H* = 0.9 eV) is illustrated in Fig. 2b by the horizontal dashed black line. 81 of the 103 fully-optimised structures are lower in enthalpy than the modified Smyth model. Additionally, a FTIR study by Jacobsen *et al.*,^36^ based on wadsleyite with a hydration level of ∼1 wt%, only found evidence of Mg3 vacancies. Further studies corroborate the present findings in terms of the sites at which protonation is likely to occur. X-ray diffraction studies by Kudoh *et al.* suggested protonation of the O1 site, however with the hydroxyl orientation disordered over O1···O1, O1···O3 and O1···O4 vectors.^31^ Recent work by Deon *et al.*,^37^ predominantly based on FTIR spectroscopy, found that the O1 and O3 sites in the vacant Mg3-centered octahedron were the favoured sites of protonation. In a ^1^H MAS NMR and FTIR study of wadsleyite at low levels of hydration (<1.5 wt%), Kohn *et al.* ^35^ found O1 protonation to be the dominant environment, while the lowest degree of protonation was associated with the O3 and O4 sites.

### 2. Comparison with NMR experiments

**Fig. 4:**
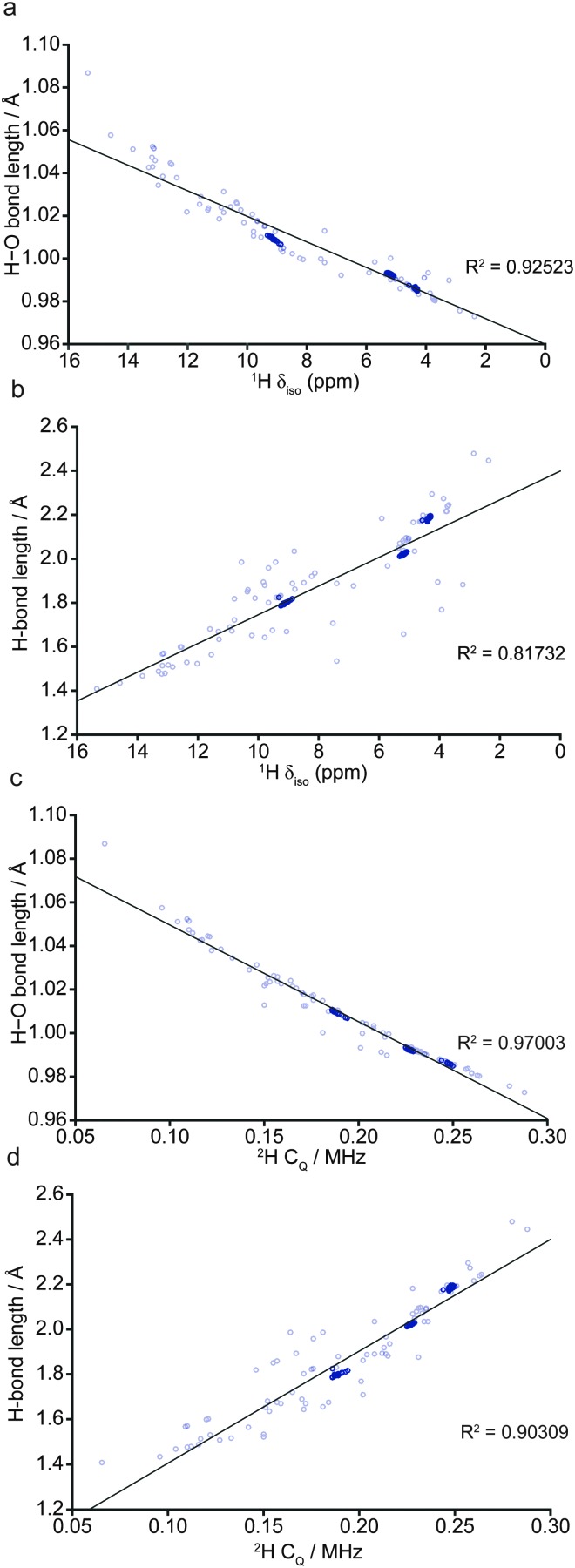
Plots of calculated covalent H–O bond length against (a) ^1^H *δ*_iso_ and (c) ^2^H *C*_*Q*_, and calculated OH (*i.e.*, hydrogen bond length) against (b) ^1^H *δ*_iso_ and (d) ^2^H *C*_*Q*_ for all 103 fully-optimised AIRSS-generated structures of hydrous wadsleyite, with points corresponding to the 64 lowest energy models shown in dark blue.

**Fig. 5:**
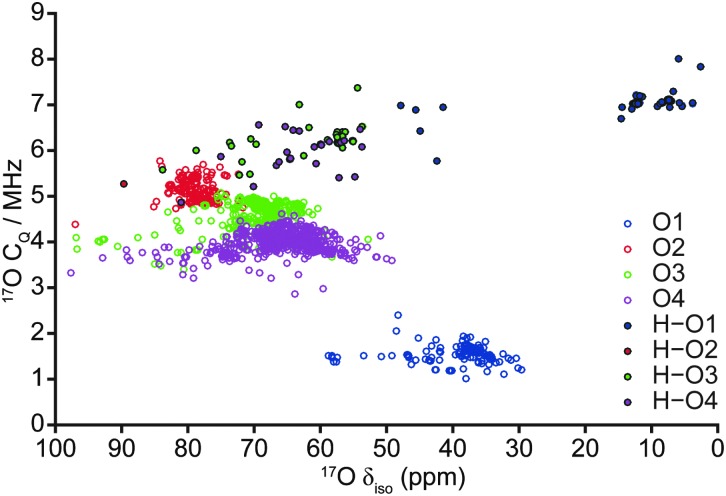
Plot of calculated ^17^O *C*_*Q*_ correlated against ^17^O *δ*_iso_ for all 103 fully-optimised AIRSS-generated structures of hydrous wadsleyite.

**Fig. 6:**
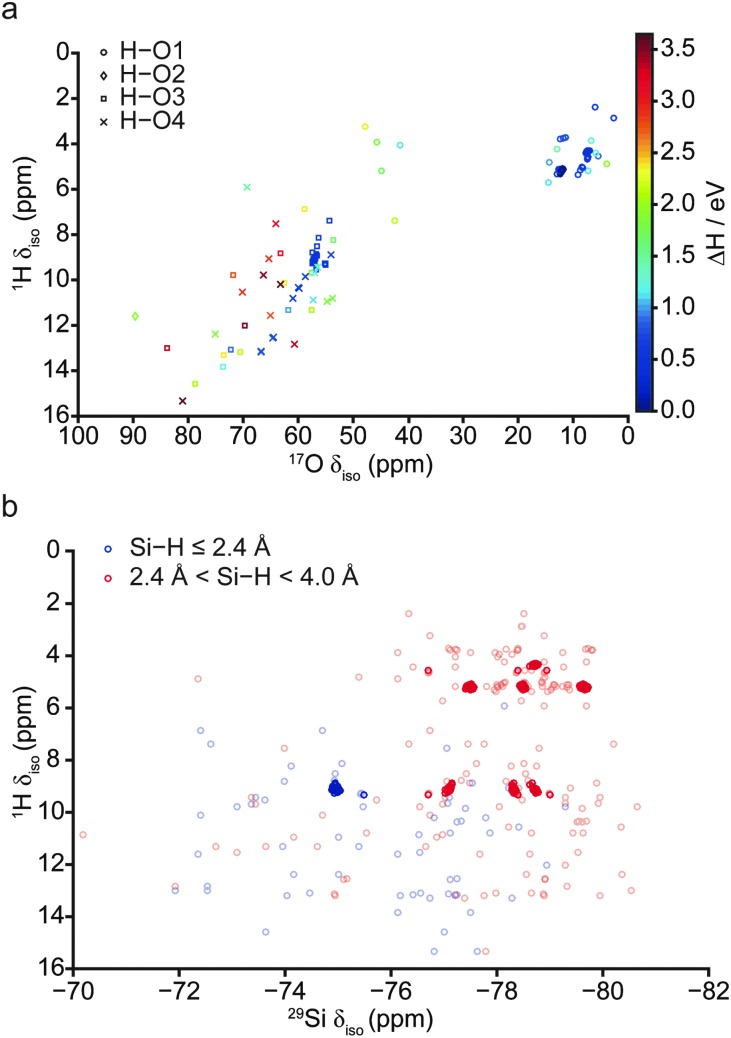
(a) Plot of calculated ^1^H/^17^O *δ*_iso_ for all 103 fully-optimised AIRSS-generated structures of hydrous wadsleyite, denoted by the site of protonation and coloured according to their relative enthalpy. (b) Plot of calculated ^1^H/^29^Si *δ*_iso_ for all fully-optimised AIRSS-generated structures of hydrous wadsleyite, distinguished by the HSi interatomic distance. The 64 structures with lowest enthalpy are shown by the darker colours.

In order to understand the implications of these results for the comparison with previous experimental NMR studies, which investigated wadsleyite with a range of hydration levels,^35,40^ multinuclear NMR parameters for all 103 fully-optimised structures were determined through GIPAW calculations^6^ in CASTEP. To facilitate this comparison, in the following, we present plots of computed bond lengths against NMR parameters (Fig. 4) and comparing different NMR parameters (Fig. 5 and 6) with chemical shift consistently on the *x*-axis. Fig. 4 shows the variation in the ^1^H isotropic shift, *δ*_iso_, and ^2^H *C*_*Q*_ with the hydroxyl (H–O) and hydrogen-bond (OH) distances. When all 103 structures are considered, Fig. 4a shows there is a strong linear correlation between the ^1^H *δ*_iso_ and the covalent H–O bond length, with a downfield shift (*i.e.*, to higher *δ*) observed with an increase in interatomic distance. However, when only structures with low Δ*H* (<0.52 eV; encompassing O1–H/O1–H and the most stable O1–H/Si–OH structures) are considered, three distinct clusters of points at *ca.* 4, 5 and 9 ppm become apparent (64 structures; represented by dark blue circles). The points at approximately 5 ppm correspond to structures with the lowest Δ*H*, where protonation occurs exclusively at the O1 sites, while the two remaining clusters, at ∼4 and ∼9 ppm, result from protonated O1 and O3 sites, respectively, in less stable structures, represented by motif **B** (see Fig. 3). As a result of the significant differences in average hydroxyl bond length (O3–H > O1–H), there is a corresponding change in ^1^H *δ*_iso_, allowing differentiation between O1–H and O3–H hydroxyls. Indeed, such high chemical shifts as predicted for O3–H were observed experimentally,^40^ and considered to be due to the relatively strong hydrogen bonds associated with silanol groups.

The computed chemical shift values for these three clusters of points do not overlay exactly with the peaks observed in the experimental ^1^H MAS NMR spectrum of hydrous wadsleyite reported previously.^40^ However, the sample studied experimentally had ∼3% H_2_O, and it has been suggested that the preferred protonation sites vary with hydration level,^35^ and small errors owing to the DFT exchange–correlation functional and referencing cannot be ruled out. It should also be noted that although the periodic approach employed here may result in small changes in structure (and therefore shift) when compared with an isolated defect or more disordered structures, attempting to include this possibility in the AIRSS approach would lead to an unfeasibly large number of possible structures that could not realistically be considered on a reasonable timescale (but would provide a challenge for further future study). However, the relative calculated isotropic shifts are in reasonably good agreement with experimental measurements, suggesting that both O1/O1 and O1/O3 substitution occur, and the calculations confirm that the previous assignments of Mg–OH and Si–OH species are correct.^40^

Fig. 4b, which shows the variation of the ^1^H *δ*_iso_ with H···O distance, also shows three distinct clusters of points when only structures with low Δ*H* are included, although noticeably more scatter is present. Structures with particularly short hydrogen bonds exhibit high ^1^H *δ*_iso_, showing that as the H–O and OH distances become similar, the proton is shifted downfield, a phenomenon that is well documented in the literature.^47–49^ *C*_*Q*_ demonstrates an almost linear dependence on the O–H bond length (Fig. 4c).

Despite the good overall agreement, five points lie noticeably below the trend line. These points correspond to structures containing protonated O1 sites where the hydroxyl bond vector is orientated away from the Mg3 vacancy (see ESI,† Fig. S2.2), in contrast to all other structures where the O1 hydroxyl vectors are co-linear with the O1···O4 octahedron edge (see Fig. 3). However, due to their high relative enthalpies (Δ*H* = 1.1 to 1.8 eV) such O1–H orientations are unlikely. The outlying point with the longest hydroxyl bond length and smallest *C*_*Q*_, 1.09 Å and 0.07 MHz, respectively, corresponds to the least stable structure found (Δ*H* = 3.7 eV). In this structure, both hydrogen atoms are located outside the Mg3 vacant octahedron. The small computed *C*_*Q*_ may be due to one proton experiencing a relatively symmetrical, though unstable, local environment. As seen in the *δ*_iso_ data, the correlation between *C*_*Q*_ and O–H bond length is noticeably stronger than that observed when the H···O hydrogen bond length is considered (Fig. 4d). Generally, *C*_*Q*_ appears to increase with decreasing O–H bond length, corresponding to the loss of symmetry as the O–H and H···O bonds become more significantly different.

Despite the calculated ^1^H and ^2^H NMR parameters providing insight into the types of hydroxyls present in a given structure (*i.e.*, Mg–OH or Si–OH), given the chemical similarity between the O3 and O4 non-bridging oxygen atoms in particular, this information is not sufficient to unequivocally determine the exact site of protonation, even when ^1^H isotropic chemical shift is plotted directly against ^2^H *C*_*Q*_ (see ESI,† Fig. S5.1). In an attempt to clarify this ambiguity, ^17^O NMR parameters were also considered, as these are known to exhibit considerable change upon protonation.^40,50^ A plot of ^17^O *C*_*Q*_ against *δ*_iso_, with points coloured according to the crystallographic O site notation, and those denoting protonated O–H species distinguished by filled circles shown in Fig. 5. Regardless of oxygen site, hydroxyl oxygen centres exhibit an increased *C*_*Q*_ (by *ca.* 2–5 MHz) relative to their non-protonated counterparts. The most significant changes are observed for O1, which also sees a large upfield shift in *δ*_iso_ (^17^O1: *δ*_iso_ ≈ 30–60 ppm, *C*_*Q*_ ≈ 1–2 MHz; ^17^O1–H: *δ*_iso_ ≈ 0–50 ppm, *C*_*Q*_ ≈ 5–8 MHz). Protonated O1–H sites further divide into two groups with *δ*_iso_ ≈ 0–20 ppm and 40–50 ppm, the latter group consisting of the five structures destabilised due to unusual orientations; these correspond to the same structures found to be outlying previously in terms of O–H and H···O distances (Fig. 4), suggesting that the ^17^O *δ*_iso_ is more sensitive to the nature of the hydroxyl group than the ^1^H *δ*_iso_. Fig. 5 illustrates that a combination of ^17^O *C*_*Q*_ and *δ*_iso_ is again sufficient to differentiate between Mg–OH and Si–OH groups, and provides support for the tentative assignment, to silanol groups, of the ^17^O signals observed at higher *δ*_iso_ experimentally.^40^ Unfortunately, ranges in silanol ^17^O NMR parameters show significant overlap. However, non-protonated silicate oxygen centres exhibit more distinct NMR parameters; in particular, O2 exhibits a relatively large *C*_*Q*_ that is typically associated with bridging oxygen species.^51^

Since the ^1^H and ^17^O NMR parameters were found to be equally sensitive indicators of the protonation site and hydroxyl orientation, variations of ^1^H *δ*_iso_ against ^17^O *δ*_iso_ are shown in Fig. 6a, focussing on structures with H–O distances ≤1.10 Å. Points are denoted by protonation site and coloured according to their relative energy. Ranges in ^1^H and ^17^O *δ*_iso_ found in this *ab initio* study are larger than those observed experimentally.^40^ However, when relative enthalpy is taken into account (by only considering structures with Δ*H* < 1.5 eV), these ranges are narrowed significantly. This suggests that experimental spectra are unlikely to display significantly intense features at *δ*_iso_ > ∼10 and ∼60 ppm in ^1^H and ^17^O NMR spectra respectively. From Fig. 6a, extensive overlap in the ranges of *δ*_iso_ observed for both O3 and O4 sites is apparent. This suggests that neither ^1^H nor ^17^O chemical shifts (nor their combination) are suitably discriminatory for a clear distinction between protonated O3 and O4 sites.

Fig. 6b shows a plot of ^1^H *vs.* ^29^Si *δ*_iso_. The values are coloured according to Si···H interatomic distance to distinguish Si–OH (blue) and Si···HO (red) interactions, where the latter includes Si centres neighbouring either silanol or O1–H species. While this plot exhibits extensive scatter, the 64 most stable structures, shown in darker colours, are found to form distinct clusters. The three clusters of points with ^1^H *δ*_iso_ ≈ 5 ppm and ^29^Si *δ*_iso_ ≈ –77.5, –78.5 and –79.5 ppm correspond to structural motifs of type **A** (Fig. 3), where protonated O1–H hydroxyls interact with three inequivalent ^29^Si nuclei with Si···H distances between 2.4 and 4.0 Å. There are numerous clusters of points corresponding to structures containing protonated O1 and O3 sites (motif **B**, Fig. 3), with the points at ^1^H *δ*_iso_ ≈ 4 ppm corresponding to the O1 hydroxyls of these structures. The feature at ∼4 ppm is made up of two clusters of points, with the same *δ*_iso_ but different HSi interatomic distances, 2.9 and 3.5 Å, respectively. The blue points at ^1^H *δ*_iso_ ≈ 9 ppm and ^29^Si *δ*_iso_ ≈ 75 ppm correspond to protonated O3 sites, indicating that as the H···Si interatomic distance decreases, both hydrogen and silicon exhibit a downfield shift, in good agreement with previous work by Griffin *et al.* ^40^ The remaining clusters at ^1^H *δ*_iso_ ≈ 9 ppm and ^29^Si *δ*_iso_ ≈ 77.0, 78.3 and 78.7 ppm represent interactions between silanol protons and ^29^Si nuclei belonging to other pyrosilicate units. This correlation was not observed experimentally,^40^ though this could be due to differences in relaxation parameters for different hydroxyl environments (*i.e.*, Mg–OH or Si–OH). Indeed, there is evidence in the literature to suggest that silanol environments in minerals are often underrepresented due to fast *T*_1*ρ*_ relaxation.^51,52^

## Conclusions

Using a novel adaptation of the recently-introduced AIRSS approach we have considered potential structural motifs in hydrous wadsleyite, a high-pressure mineral of importance in the Earth between depths of ∼410 and 530 km. Rather than starting with an empty cell (as in the original AIRSS method), a Mg^2+^ vacancy was created in an initial structural model of anhydrous wadsleyite and two H^+^ then randomly inserted, before optimisation using DFT. This resulted in 819 initial structures, of which a subset of 103 structures were subjected to high accuracy geometry optimisation and used to predict NMR parameters. The most stable structures feature two protonated O1 sites, with the hydroxyls aligned in hydrogen-bonding environments parallel to the O1···O4 edge of the vacant octahedron, in agreement with Deon *et al.* ^37^ An increase in enthalpy is observed for structures that contain one silanol hydroxyl group, and even larger increases for models where both substituted protons are associated with the pyrosilicate oxygens. We therefore suggest a structural model in which O1 oxygens are the predominant site of protonation; although in the present periodic DFT calculations silanol group formation is found to carry a high enthalpic cost (∼0.5 eV), and the present theoretical model precludes the estimation of configurational entropy, it is thought that the presence of silanol groups as low level defects in the experimental system would lead to a significant entropic contribution (*i.e.*, a large degeneracy) that would overcome the enthalpy of their formation. Indeed, such a hierarchical structural model is corroborated by observations from FTIR, X-ray diffraction and NMR experiments.^31,35–37,40^

Despite consideration of a restricted set of models (*i.e.*, limited to Mg3 vacancies and 1.6 wt% hydration), the predicted NMR parameters agree well with previous multinuclear NMR experimental results on a sample with ∼3.0 wt% H_2_O, and are able to confirm the interpretation and assignment of a number of spectral features.^40^ The high ^1^H chemical shifts observed are shown to result from strong hydrogen bonding to nearby O species, and a linear correlation of ^1^H *δ*_iso_ and ^2^H *C*_*Q*_ with hydroxyl O–H bond length and H···O hydrogen bond length is clear. The structure proposed by Smyth (modified to 1.6 wt% hydration) has a high relative enthalpy; *ca.* 1.0 eV above the most stable structure generated by AIRSS in this work. This appears to be due to the high-symmetry point charge model adopted by Smyth, which precludes the formation of stabilising hydrogen-bonding interactions.

Further work will seek to test these conclusions in progressively more complex models. This will include the investigation of higher levels of hydration (3.3 wt%; two Mg vacancies and four H per unit cell) and the assessment of alternative vacancies of Mg1, Mg2, Si sites and their combinations (in ordered unit cells). In addition, the systematic use of supercells will assess the effect of positional disorder of identified motifs. As the complexity of models used increases, the opportunity for comparison with the true disordered system arises, and at this point, the level of theory may be tested by the inclusion of, *e.g.*, dispersion effects *via* appropriate correction schemes. Note, such steps lead to a highly combinatorial problem and the simultaneous assessment of each phenomenon is unlikely to be possible. As such, quantifying the degeneracy of any given arrangement of defects is an aim that may only be fulfilled by experiment.

The AIRSS philosophy of randomly generating candidates for unknown and disordered structures, combined with the prediction of solid-state NMR parameters (providing a direct link to experiment) heralds a step change in the characterisation of complex materials. We envisage opportunities for the application of this combinatorial approach to areas as diverse as energy materials, gas storage and separation, microporous frameworks, (supra)molecular self-assembly and catalysis.
