# Supplementary material for: Hunting for hydrogen: random structure searching and prediction of NMR parameters of hydrous wadsleyite
Source: Phys Chem Chem Phys. 2016 Mar 29;18(15):10173–81. doi: 10.1039/c6cp01529h (PMC4840454; doi:10.1039/c6cp01529h)
Supplement: Supplementary file 1 [file CP-018-C6CP01529H-s001.pdf]

Electronic Supplementary Material (ESI) for Physical Chemistry Chemical Physics.
This journal is © the Owner Societies 2016

Hunting for Hydrogen: Random Structure Searching and Prediction of NMR Parameters of Hydrous Wadsleyite

Robert F. Moran, David McKay, Chris J. Pickard, Andrew J. Berry, John M. Griffin and Sharon E. Ashbrook*

Supporting Information

S1. Generation of structural models using AIRSS

S2. High-lying structures

S3. Supercell calculations

S4. Modified Smyth model

S5. ^{1}H δ_{iso} *vs* ^{2}H C_Q

S6. Total enthalpies of computed structures

S1. Generation of structural models using AIRSS

The AIRSS input CELL file is shown below. The line "`H 0.25… 0.25… 0.75… # H % NUM=2 POSAMP=3`" instructs AIRSS to place two H atoms at the site of a Mg3 vacancy and move each of them by a random distance (of maximum 3 Å). The global "`#POSAMP=0`" ensures the positions of the other atoms do not change, and "`#MINSEP=0.75`" ensures dihydrogen molecules are not generated.

AIRSS CELL file:

```
%BLOCK LATTICE_CART
      11.460000000000022       0.000000000000000       0.000000000000000
       0.000000000000000       5.692100000000002       0.000000000000000
       0.000000000000000       0.000000000000000       8.253000000000004
#FIX
%ENDBLOCK LATTICE_CART
FIX_ALL_CELL : true
%BLOCK POSITIONS_FRAC
  O   0.3729000000000000   0.9891999999999989   0.7428999999999997
  O  -0.1271000000000002   0.4891999999999988   0.2428999999999997
  O   0.6271000000000002   0.5108000000000016   0.7428999999999997
  O   0.1271000000000001   0.0108000000000012   0.2428999999999998
  O   0.6271000000000002   0.4891999999999991   0.2571000000000003
  O   0.1271000000000001   0.9891999999999989   0.7571000000000002
  O   0.3729000000000000   0.0108000000000014   0.2571000000000003
  O  -0.1271000000000002   0.5108000000000013   0.7571000000000002
  O   0.6271000000000002   0.0108000000000015   0.2571000000000003
  O   0.1271000000000001   0.5108000000000013   0.7571000000000002
  O   0.3729000000000000   0.4891999999999989   0.2571000000000003
  O  -0.1271000000000002   0.9891999999999989   0.7571000000000002
  O   0.3729000000000000   0.5108000000000016   0.7428999999999999
  O  -0.1271000000000002   0.0108000000000014   0.2428999999999998
  O   0.6271000000000002   0.9891999999999992   0.7428999999999999
  O   0.1271000000000001   0.4891999999999989   0.2428999999999997
  O   0.5000000000000001   0.2500000000000002   0.9686999999999999
  O   0.5000000000000001   0.2500000000000002   0.4667999999999997
  O  -0.0000000000000000   0.7500000000000002   0.4687000000000000
  O  -0.0000000000000000   0.7500000000000002   0.9667999999999998
  O   0.5000000000000000   0.7500000000000002   0.0313000000000001
  O   0.5000000000000000   0.7500000000000002   0.5332000000000003
  O  -0.0000000000000000   0.2500000000000001   0.5313000000000001
  O  -0.0000000000000000   0.2500000000000001   0.0332000000000004
  O   0.7394999999999993   0.7500000000000003   0.5056000000000003
  O   0.2394999999999991   0.2500000000000002   0.0056000000000003
  O   0.2605000000000010   0.7500000000000002   0.5056000000000003
  O  -0.2394999999999992   0.2499999999999998   0.0056000000000002
  O   0.2605000000000010   0.2500000000000002   0.4943999999999998
  O  -0.2394999999999992   0.7500000000000001   0.9943999999999998
  O   0.7394999999999993   0.2500000000000002   0.4943999999999998
  O   0.2394999999999991   0.7500000000000001   0.9943999999999998
 H    0.2500000000000001   0.2500000000000001   0.7499999999999999 # H % NUM=2
POSAMP=3
 Mg   0.7500000000000001   0.2500000000000002   0.7500000000000001
 Mg   0.7500000000000001   0.7500000000000002   0.2500000000000001
```

```
 Mg   0.2500000000000001   0.7500000000000002   0.2500000000000000
 Mg   0.0000000000000000   0.7500000000000001   0.2200999999999996
 Mg   0.5000000000000000   0.2500000000000001   0.7200999999999996
 Mg   0.0000000000000000   0.2499999999999999   0.7799000000000005
 Mg   0.5000000000000000   0.7500000000000002   0.2799000000000004
 Mg   0.3763999999999995   0.0000000000000001   0.0000000000000001
 Mg   0.6236000000000007   0.5000000000000001   0.0000000000000001
 Mg   0.1236000000000006   0.0000000000000001   0.5000000000000000
 Mg   0.6236000000000007   0.0000000000000003   0.0000000000000001
 Mg   0.1236000000000006   0.5000000000000000   0.5000000000000000
 Mg   0.3763999999999995   0.5000000000000003   0.0000000000000001
 Mg   0.8763999999999995   0.0000000000000003   0.5000000000000001
 Mg   0.8763999999999995   0.5000000000000004   0.5000000000000001
 Si   0.3701000000000000   0.2500000000000004   0.3665999999999987
 Si  -0.1299000000000002   0.7500000000000003   0.8665999999999986
 Si   0.6299000000000002   0.2499999999999999   0.3665999999999987
 Si   0.1299000000000001   0.7499999999999998   0.8665999999999986
 Si   0.6299000000000002   0.7500000000000003   0.6334000000000014
 Si   0.1299000000000001   0.2500000000000002   0.1334000000000015
 Si   0.3701000000000000   0.7500000000000001   0.6334000000000015
 Si  -0.1299000000000002   0.2499999999999999   0.1334000000000015
%ENDBLOCK POSITIONS_FRAC

KPOINTS_MP_SPACING 0.1
SYMMETRY_GENERATE
SNAP_TO_SYMMETRY
#POSAMP=0
#SYMMOPS=1
#CONS=0.25
#MINSEP=0.75
```

An output CELL file generated from the AIRSS process, which can be used for further CASTEP calculations, is shown below.

Generated CELL file:

```
%BLOCK LATTICE_CART
  11.460000000000022   0.000000000000000   0.000000000000000
   0.000000000000000   5.692100000000002   0.000000000000000
   0.000000000000001   0.000000000000001   8.253000000000004
%ENDBLOCK LATTICE_CART

%BLOCK POSITIONS_FRAC
   O   0.372900000000000   0.989199999999999   0.742899999999999
   O   0.872900000000000   0.489199999999999   0.242900000000000
   O   0.627100000000000   0.510800000000002   0.742899999999999
   O   0.127100000000000   0.010800000000001   0.242900000000000
   O   0.627100000000000   0.489199999999999   0.257100000000000
   O   0.127100000000000   0.989199999999999   0.757100000000000
   O   0.372900000000000   0.010800000000001   0.257100000000000
   O   0.872900000000000   0.510800000000001   0.757100000000000
   O   0.627100000000000   0.010800000000001   0.257100000000000
   O   0.127100000000000   0.510800000000001   0.757100000000000
   O   0.372900000000000   0.489199999999999   0.257100000000000
   O   0.872900000000000   0.989199999999999   0.757100000000000
   O   0.372900000000000   0.510800000000002   0.742900000000000
   O   0.872900000000000   0.010800000000001   0.242900000000000
   O   0.627100000000000   0.989199999999999   0.742900000000000
```

```
   O   0.127100000000000   0.489199999999999   0.242900000000000
   O   0.500000000000000   0.250000000000000   0.968700000000000
   O   0.500000000000000   0.250000000000000   0.466800000000000
   O   0.000000000000000   0.750000000000000   0.468700000000000
   O   0.000000000000000   0.750000000000000   0.966799999999999
   O   0.500000000000000   0.750000000000000   0.031300000000000
   O   0.500000000000000   0.750000000000000   0.533200000000000
   O   1.000000000000000   0.250000000000000   0.531300000000000
   O   1.000000000000000   0.250000000000000   0.033200000000000
   O   0.739499999999999   0.750000000000000   0.505600000000000
   O   0.239499999999999   0.250000000000000   0.005600000000000
   O   0.260500000000001   0.750000000000000   0.505600000000000
   O   0.760500000000001   0.250000000000000   0.005600000000000
   O   0.260500000000001   0.250000000000000   0.494400000000000
   O   0.760500000000001   0.750000000000000   0.994400000000000
   O   0.739499999999999   0.250000000000000   0.494400000000000
   O   0.239499999999999   0.750000000000000   0.994400000000000
   H   0.192711168454776   0.786679356198195   0.849206932585415
   H   0.413222667096391   0.081108848047837   0.858053016559826
  Mg   0.750000000000000   0.250000000000000   0.750000000000000
  Mg   0.750000000000000   0.750000000000000   0.250000000000000
  Mg   0.250000000000000   0.750000000000000   0.250000000000000
  Mg   1.000000000000000   0.750000000000000   0.220100000000000
  Mg   0.500000000000000   0.250000000000000   0.720099999999999
  Mg   0.000000000000000   0.250000000000000   0.779900000000000
  Mg   0.500000000000000   0.750000000000000   0.279900000000000
  Mg   0.376399999999999   0.000000000000000   0.000000000000000
  Mg   0.623600000000001   0.500000000000000   0.000000000000000
  Mg   0.123600000000001   0.000000000000000   0.500000000000000
  Mg   0.623600000000001   0.000000000000000   0.000000000000000
  Mg   0.123600000000001   0.500000000000000   0.500000000000000
  Mg   0.376399999999999   0.500000000000000   0.000000000000000
  Mg   0.876399999999999   0.000000000000000   0.500000000000000
  Mg   0.876399999999999   0.500000000000001   0.500000000000000
  Si   0.370100000000000   0.250000000000000   0.366599999999999
  Si   0.870100000000000   0.750000000000000   0.866599999999998
  Si   0.629900000000000   0.250000000000000   0.366599999999999
  Si   0.123807630730438   0.746442285854145   0.868287040569803
  Si   0.629900000000000   0.750000000000000   0.633400000000001
  Si   0.129900000000000   0.250000000000000   0.133400000000001
  Si   0.370100000000000   0.750000000000000   0.633400000000001
  Si   0.870100000000000   0.250000000000000   0.133400000000001
%ENDBLOCK POSITIONS_FRAC
```

S2. High-lying structures

High-enthalpy structures were found to include those containing more than one silanol (Si–OH) group, O2 hydroxyls, or O1 hydroxyls orientated away from the Mg3 vacancy. From the 103 fully optimised structures, only one was found to contain an O2 hydroxyl (see Figure S2.1), while a total of five were found to contain unfavourably oriented O1 hydroxyls (see Figure S2.2).

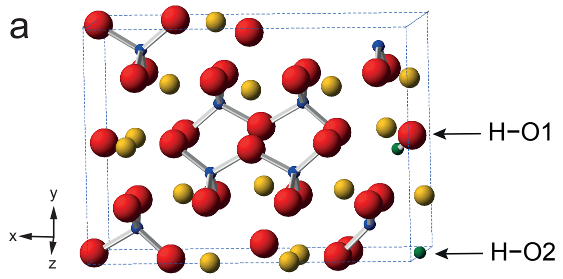

Figure S2.1: (a) Schematic showing the unit cell of the one structure (ranked 93/103) found to contain an O2 hydroxyl, (b) expansion of the O2 hydroxyl environment, with hydrogen, silicon, magnesium and oxygen shown in green, blue, yellow and red, respectively.

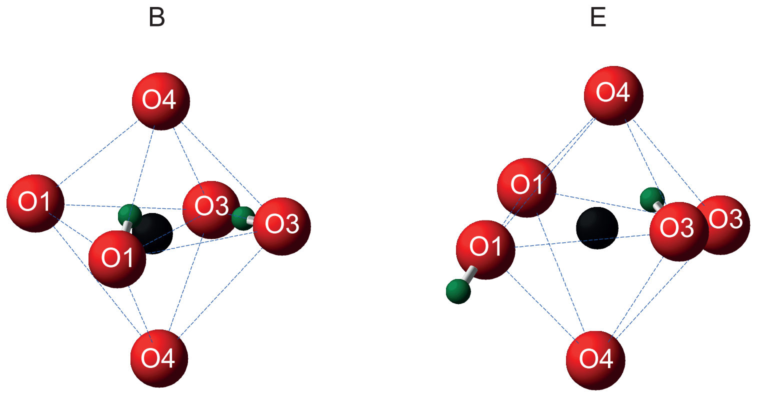

Figure S2.2: Schematic showing an O1 hydroxyl pointing towards (**B**) and away from (**E**) the Mg3 vacancy. For structures **A-D** see main text.

.

S3. Supercell calculations

Supercell structures were generated based on the most stable fully-optimised AIRSS generated structure (structure **A**, see main text) as 2 × 2 × 1 supercells. A geometry optimisation was then performed on all atomic positions with the lattice parameters fixed. These resulted in structures with 4 × Mg3 vacancies and 8 × H atoms. The unchanged structure (*i.e.*, with 8 × O1–H hydroxyls) provided a new enthalpy minimum (supercell **I**, Figure S3.1). For supercell **II** one H was moved to give an O3–H hydroxyl group (*cf.* **B**), giving 1 × O3–H and 7 × O1–H hydroxyls. Supercell **III** similarly has a single O4–H and 7 × O1–H hydroxyls (*cf.* **C**). In such a model, the ratio of "defect" hydroxyls to hydroxyls bound to O1 is 1:7, rather than 1:1 as required in unit cell models. Supercell **IV** contains 8 × O1–H hydroxyls, equivalent to **I**, showing that increasing the system size allows the Mg3 vacancies (and corresponding protons) to be spread about the system, increasing separation of like charges; an arrangement not available in the unit cell.

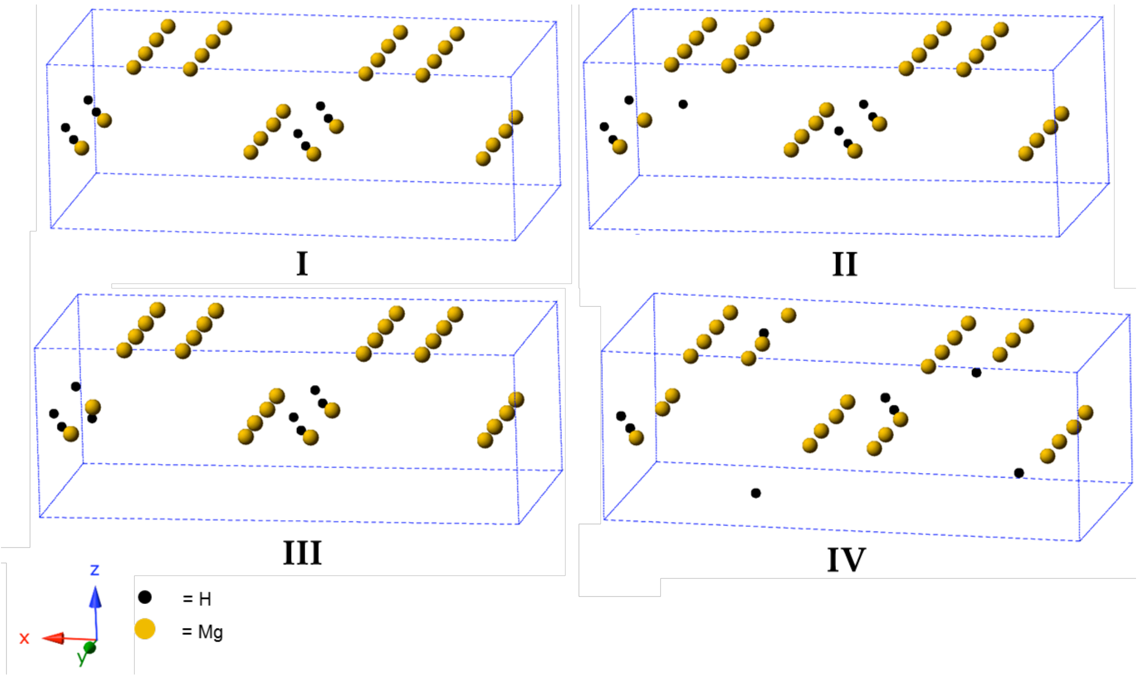

Figure S3.1: Optimised 2 × 2 × 1 supercells **I**, based on **A**; **II**, with a single O3–H hydroxyl (as found in **B**); **III**, with a single O4–H hydroxyl (as found in **C**) and **IV**, with Mg3 vacancies and 8 × O1–H hydroxyls spread to maximise separation of like charges. Si, O, Mg1 and Mg2 atoms omitted to allow focus on Mg3 atoms (yellow) and H (black).

The same change is induced going from **I** to **II** to **III** as from **A** to **B** to **C**, in that a proton is moved from a favourable O1–H···O4 environment to a less favourable position. Therefore, relative enthalpies are equivalent (with slight differences owing to the increased flexibility of the supercell system) at ΔH = 0.00, 0.47 and 0.56 eV for **I**, **II** and **III** respectively (Table S3.1). However, taking into account the change per formula unit with the 4-fold increase in system size from $Mg_{12}Si_8O_{32}H_2$ to $Mg_{60}Si_{32}O_{128}H_8$ gives ΔH = 0.00, 0.12 and 0.14 eV/unit. As such the increased positional entropy available to the supercell system alleviates some of the enthalpy cost of forming a less stable H environment. Therefore O3–H and O4–H hydroxyl groups are considered to form as defects while O1–H hydroxyls predominate. In the case of supercell **IV**, the increased flexibility of the supercell approach has allowed for an improvement due to greater spread of Mg3 vacancies, giving ΔH = –0.14 eV/unit (*i.e., below* **I**).

Table S3.1: Comparison of relative enthalpies (ΔH) of supercells showing the reduction in ΔH as O3–H and O4–H hydroxyls are treated as defects and as Mg3 vacancies are spread in the supercell.

Structure	ΔH / eV	ΔH/4[a] / eV
**I**	0.00	0.00
**II**	0.47	0.12
**III**	0.56	0.14
**IV**	–0.56	–0.14

[a] *i.e.*, corresponding to the per formula unit increase in the supercell structures.

S4. Modified Smyth model

The original Smyth structure represented a fully hydrated (~3.3 wt%) system, thus requiring some modification before it could be compared to the AIRSS-generated structures, which were based on a ~1.6 wt% hydration level. The modification simply involved replacing two protons with a magnesium on an Mg2 site, leaving two hydroxyls aligned parallel to the *z*-axis in close proximity to the remaining Mg2 vacancy, as shown in Figure S4.1. This structure was then geometry optimised to calculate the relative enthalpy.

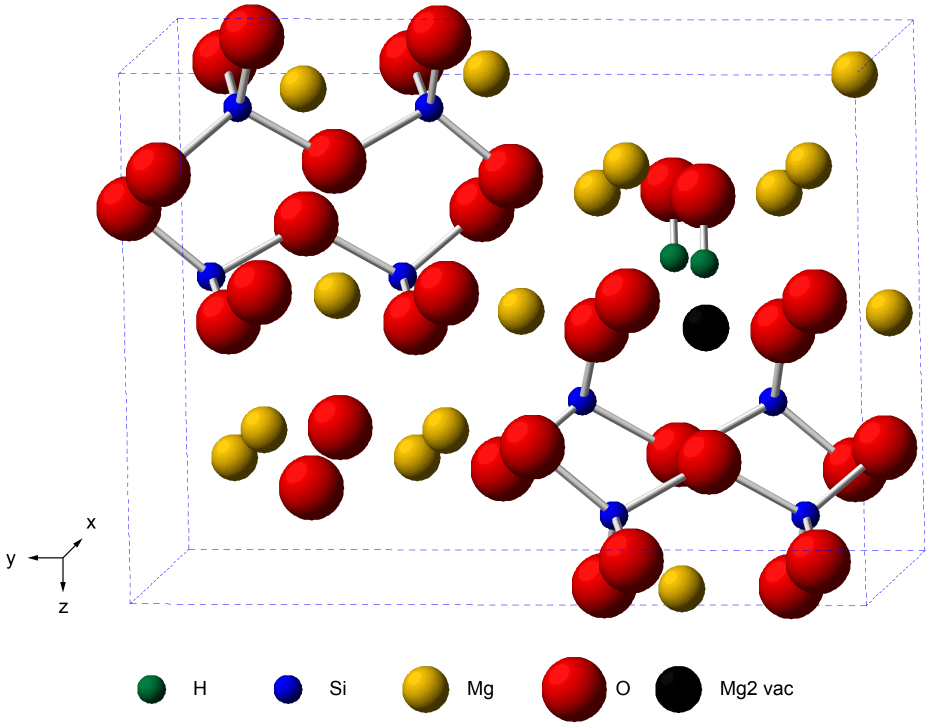

Figure S4.1: Smyth structure modified to result in a ~1.6 wt% hydration level.

S5. ^{1}H δ_{iso} *vs* ^{2}H C_Q

A plot of ^{2}H C_Q against ^{1}H δ_{iso} is shown in Figure S5.1. It shows that, although O1 hydroxyls can be separated from silanol hydroxyls using a combination of ^{1}H δ_{iso} and ^{2}H C_Q, it is much more difficult to differentiate between the crystallographically-similar pyrosilicate oxygen sites, particularly the O3 and O4.

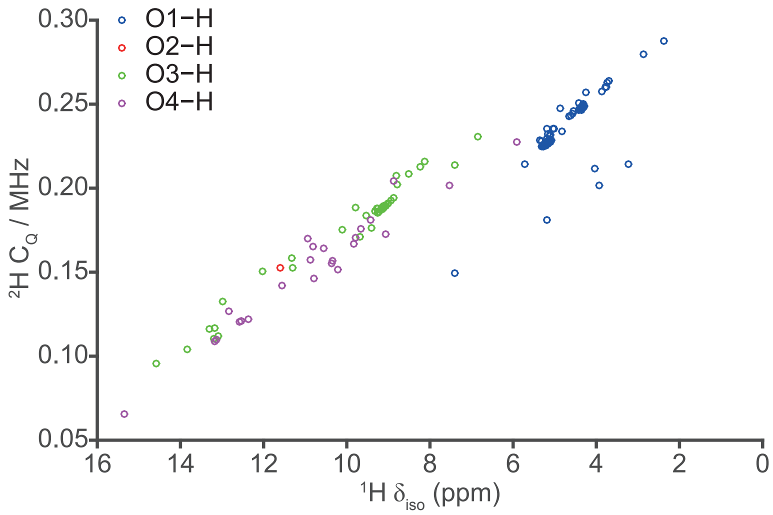

Figure S5.1: Plot of calculated ^{2}H C_Q against ^{1}H δ_{iso} for all 103 fully-optimised AIRSS-generated structures of hydrous wadsleyite.

S6. Total enthalpies of computed structures

Table S6.1: Total enthalpies of AIRSS-generated structures (see Figure 2).

Index	Enthalpy / eV
1	–29557.6020
2	–29557.6017
3	–29557.6000
4	–29557.5966
5	–29557.5956
6	–29557.5949
7	–29557.5927
8	–29557.5926
9	–29557.5918
10	–29557.5901
11	–29557.5895
12	–29557.5877
13	–29557.5876
14	–29557.5873
15	–29557.5860
16	–29557.5845
17	–29557.5842
18	–29557.5818
19	–29557.5816
20	–29557.5814
21	–29557.5813
22	–29557.5812
23	–29557.5803
24	–29557.5800
25	–29557.5795
26	–29557.5792
27	–29557.5790
28	–29557.5777
29	–29557.5767
30	–29557.5766
31	–29557.5757
32	–29557.5754
33	–29557.5752
34	–29557.5752
35	–29557.5748
36	–29557.5741
37	–29557.5737
38	–29557.5733
39	–29557.5730
40	–29557.5728
41	–29557.5727
42	–29557.5719
43	–29557.5719
44	–29557.5714
45	–29557.5711
46	–29557.5710
47	–29557.5707
48	–29557.5706
49	–29557.5705
50	–29557.5702
51	–29557.5702
52	–29557.5697
53	–29557.5697
54	–29557.5695
55	–29557.5695
56	–29557.5688
57	–29557.5686
58	–29557.5684
59	–29557.5676
60	–29557.5676
61	–29557.5675
62	–29557.5671
63	–29557.5668
64	–29557.5667
65	–29557.5664
66	–29557.5655
67	–29557.5653
68	–29557.5652
69	–29557.5650
70	–29557.5646
71	–29557.5646
72	–29557.5642
73	–29557.5642
74	–29557.5640
75	–29557.5637
76	–29557.5635
77	–29557.1435
78	–29557.1401
79	–29557.1378
80	–29557.1366
81	–29557.1339
82	–29557.1335
83	–29557.1334
84	–29557.1318
85	–29557.1304
86	–29557.1299
87	–29557.1253
88	–29557.1251
89	–29557.1235
90	–29557.1230
91	–29557.1222
92	–29557.1207
93	–29557.1195
94	–29557.1174
95	–29557.1167
96	–29557.1164
97	–29557.1163
98	–29557.1161
99	–29557.1156
100	–29557.1130
101	–29557.1102
102	–29557.1081
103	–29557.1067
104	–29557.1060
105	–29557.1059
106	–29557.1038
107	–29557.1015
108	–29557.1014
109	–29557.1014
110	–29557.1011
111	–29557.0997
112	–29557.0989
113	–29557.0985
114	–29557.0967
115	–29557.0965
116	–29557.0962
117	–29557.0959
118	–29557.0958
119	–29557.0958
120	–29557.0956
121	–29557.0942
122	–29557.0939
123	–29557.0937
124	–29557.0934
125	–29557.0931
126	–29557.0930
127	–29557.0929
128	–29557.0922

129	–29557.0917	176	–29557.0772	223	–29557.0639
130	–29557.0916	177	–29557.0771	224	–29557.0629
131	–29557.0908	178	–29557.0770	225	–29557.0622
132	–29557.0895	179	–29557.0769	226	–29557.0621
133	–29557.0893	180	–29557.0768	227	–29557.0614
134	–29557.0883	181	–29557.0767	228	–29557.0613
135	–29557.0878	182	–29557.0765	229	–29557.0598
136	–29557.0878	183	–29557.0765	230	–29557.0596
137	–29557.0877	184	–29557.0760	231	–29557.0595
138	–29557.0873	185	–29557.0759	232	–29557.0592
139	–29557.0872	186	–29557.0759	233	–29557.0591
140	–29557.0865	187	–29557.0758	234	–29557.0578
141	–29557.0864	188	–29557.0754	235	–29557.0577
142	–29557.0863	189	–29557.0750	236	–29557.0576
143	–29557.0863	190	–29557.0747	237	–29557.0573
144	–29557.0862	191	–29557.0747	238	–29557.0565
145	–29557.0861	192	–29557.0747	239	–29557.0562
146	–29557.0860	193	–29557.0744	240	–29557.0561
147	–29557.0853	194	–29557.0742	241	–29557.0560
148	–29557.0848	195	–29557.0740	242	–29557.0559
149	–29557.0839	196	–29557.0738	243	–29557.0548
150	–29557.0831	197	–29557.0738	244	–29557.0542
151	–29557.0828	198	–29557.0736	245	–29557.0539
152	–29557.0828	199	–29557.0736	246	–29557.0522
153	–29557.0822	200	–29557.0728	247	–29557.0516
154	–29557.0821	201	–29557.0725	248	–29557.0514
155	–29557.0820	202	–29557.0723	249	–29557.0512
156	–29557.0817	203	–29557.0718	250	–29557.0503
157	–29557.0816	204	–29557.0714	251	–29557.0498
158	–29557.0816	205	–29557.0714	252	–29557.0495
159	–29557.0814	206	–29557.0714	253	–29557.0480
160	–29557.0812	207	–29557.0713	254	–29557.0474
161	–29557.0810	208	–29557.0706	255	–29557.0464
162	–29557.0803	209	–29557.0701	256	–29557.0463
163	–29557.0799	210	–29557.0699	257	–29557.0459
164	–29557.0797	211	–29557.0698	258	–29557.0459
165	–29557.0794	212	–29557.0690	259	–29557.0446
166	–29557.0792	213	–29557.0683	260	–29557.0401
167	–29557.0791	214	–29557.0672	261	–29557.0387
168	–29557.0790	215	–29557.0667	262	–29557.0380
169	–29557.0786	216	–29557.0665	263	–29557.0378
170	–29557.0786	217	–29557.0662	264	–29557.0367
171	–29557.0783	218	–29557.0657	265	–29557.0364
172	–29557.0780	219	–29557.0652	266	–29557.0359
173	–29557.0778	220	–29557.0649	267	–29557.0356
174	–29557.0775	221	–29557.0644	268	–29557.0348
175	–29557.0772	222	–29557.0640	269	–29557.0341

270	–29557.0328	317	–29557.0184	364	–29557.0026
271	–29557.0322	318	–29557.0183	365	–29557.0015
272	–29557.0321	319	–29557.0183	366	–29557.0012
273	–29557.0314	320	–29557.0182	367	–29557.0010
274	–29557.0302	321	–29557.0181	368	–29557.0009
275	–29557.0300	322	–29557.0175	369	–29557.0004
276	–29557.0296	323	–29557.0171	370	–29556.9966
277	–29557.0290	324	–29557.0169	371	–29556.9964
278	–29557.0288	325	–29557.0169	372	–29556.9962
279	–29557.0284	326	–29557.0168	373	–29556.9956
280	–29557.0284	327	–29557.0167	374	–29556.9955
281	–29557.0279	328	–29557.0164	375	–29556.9954
282	–29557.0278	329	–29557.0160	376	–29556.9954
283	–29557.0270	330	–29557.0150	377	–29556.9943
284	–29557.0267	331	–29557.0147	378	–29556.9939
285	–29557.0263	332	–29557.0145	379	–29556.9931
286	–29557.0256	333	–29557.0144	380	–29556.9927
287	–29557.0256	334	–29557.0141	381	–29556.9909
288	–29557.0255	335	–29557.0132	382	–29556.9886
289	–29557.0255	336	–29557.0128	383	–29556.9885
290	–29557.0254	337	–29557.0120	384	–29556.9884
291	–29557.0242	338	–29557.0120	385	–29556.9851
292	–29557.0240	339	–29557.0119	386	–29556.9844
293	–29557.0238	340	–29557.0096	387	–29556.9837
294	–29557.0235	341	–29557.0094	388	–29556.9826
295	–29557.0232	342	–29557.0094	389	–29556.9824
296	–29557.0230	343	–29557.0086	390	–29556.9823
297	–29557.0228	344	–29557.0073	391	–29556.9818
298	–29557.0227	345	–29557.0072	392	–29556.9803
299	–29557.0226	346	–29557.0068	393	–29556.9798
300	–29557.0226	347	–29557.0063	394	–29556.9797
301	–29557.0223	348	–29557.0061	395	–29556.9793
302	–29557.0221	349	–29557.0060	396	–29556.9793
303	–29557.0218	350	–29557.0059	397	–29556.9788
304	–29557.0218	351	–29557.0057	398	–29556.9785
305	–29557.0217	352	–29557.0052	399	–29556.9782
306	–29557.0213	353	–29557.0050	400	–29556.9779
307	–29557.0207	354	–29557.0045	401	–29556.9778
308	–29557.0206	355	–29557.0045	402	–29556.9774
309	–29557.0203	356	–29557.0044	403	–29556.9770
310	–29557.0202	357	–29557.0039	404	–29556.9770
311	–29557.0200	358	–29557.0038	405	–29556.9769
312	–29557.0200	359	–29557.0033	406	–29556.9768
313	–29557.0191	360	–29557.0032	407	–29556.9766
314	–29557.0190	361	–29557.0031	408	–29556.9761
315	–29557.0188	362	–29557.0030	409	–29556.9757
316	–29557.0185	363	–29557.0030	410	–29556.9754

411	–29556.9750	458	–29556.8250	505	–29556.8013
412	–29556.9747	459	–29556.8245	506	–29556.8013
413	–29556.9746	460	–29556.8228	507	–29556.8010
414	–29556.9738	461	–29556.8226	508	–29556.8010
415	–29556.9728	462	–29556.8217	509	–29556.8008
416	–29556.9718	463	–29556.8207	510	–29556.8000
417	–29556.9718	464	–29556.8202	511	–29556.7998
418	–29556.9706	465	–29556.8191	512	–29556.7994
419	–29556.9706	466	–29556.8185	513	–29556.7987
420	–29556.9698	467	–29556.8176	514	–29556.7982
421	–29556.9696	468	–29556.8166	515	–29556.7981
422	–29556.9694	469	–29556.8160	516	–29556.7979
423	–29556.9693	470	–29556.8151	517	–29556.7977
424	–29556.9676	471	–29556.8147	518	–29556.7971
425	–29556.9673	472	–29556.8144	519	–29556.7970
426	–29556.9670	473	–29556.8136	520	–29556.7967
427	–29556.9656	474	–29556.8116	521	–29556.7966
428	–29556.9640	475	–29556.8109	522	–29556.7964
429	–29556.9639	476	–29556.8097	523	–29556.7958
430	–29556.9637	477	–29556.8097	524	–29556.7955
431	–29556.9634	478	–29556.8096	525	–29556.7953
432	–29556.9611	479	–29556.8095	526	–29556.7941
433	–29556.9609	480	–29556.8094	527	–29556.7906
434	–29556.9602	481	–29556.8092	528	–29556.7871
435	–29556.9565	482	–29556.8088	529	–29556.7870
436	–29556.9558	483	–29556.8087	530	–29556.7859
437	–29556.9555	484	–29556.8082	531	–29556.7748
438	–29556.9500	485	–29556.8081	532	–29556.7696
439	–29556.9470	486	–29556.8076	533	–29556.7671
440	–29556.9380	487	–29556.8064	534	–29556.7122
441	–29556.9376	488	–29556.8063	535	–29556.7056
442	–29556.9268	489	–29556.8062	536	–29556.7008
443	–29556.9263	490	–29556.8059	537	–29556.6957
444	–29556.9260	491	–29556.8052	538	–29556.6570
445	–29556.9219	492	–29556.8051	539	–29556.6518
446	–29556.9214	493	–29556.8048	540	–29556.6470
447	–29556.9205	494	–29556.8047	541	–29556.6425
448	–29556.9159	495	–29556.8041	542	–29556.6418
449	–29556.8469	496	–29556.8035	543	–29556.6400
450	–29556.8388	497	–29556.8032	544	–29556.6387
451	–29556.8350	498	–29556.8024	545	–29556.6342
452	–29556.8333	499	–29556.8024	546	–29556.6239
453	–29556.8312	500	–29556.8024	547	–29556.6045
454	–29556.8301	501	–29556.8023	548	–29556.6005
455	–29556.8290	502	–29556.8019	549	–29556.5354
456	–29556.8265	503	–29556.8019	550	–29556.5301
457	–29556.8263	504	–29556.8018	551	–29556.5264

552	–29556.5263	599	–29556.3780	646	–29556.2412
553	–29556.5249	600	–29556.3777	647	–29556.2400
554	–29556.5202	601	–29556.3776	648	–29556.2385
555	–29556.5158	602	–29556.3775	649	–29556.2376
556	–29556.5145	603	–29556.3750	650	–29556.2367
557	–29556.5120	604	–29556.3740	651	–29556.2347
558	–29556.5104	605	–29556.3738	652	–29556.2342
559	–29556.5104	606	–29556.3736	653	–29556.2311
560	–29556.5099	607	–29556.3211	654	–29556.2279
561	–29556.5098	608	–29556.3005	655	–29556.2142
562	–29556.5068	609	–29556.2979	656	–29556.2128
563	–29556.5058	610	–29556.2960	657	–29556.2058
564	–29556.5044	611	–29556.2941	658	–29556.1428
565	–29556.5036	612	–29556.2941	659	–29556.1404
566	–29556.5014	613	–29556.2895	660	–29556.1396
567	–29556.5000	614	–29556.2859	661	–29556.1370
568	–29556.4999	615	–29556.2849	662	–29556.1332
569	–29556.4997	616	–29556.2840	663	–29556.1152
570	–29556.4949	617	–29556.2836	664	–29556.1141
571	–29556.4949	618	–29556.2819	665	–29556.1136
572	–29556.4941	619	–29556.2802	666	–29556.1105
573	–29556.4922	620	–29556.2799	667	–29556.1079
574	–29556.4911	621	–29556.2798	668	–29556.1074
575	–29556.4904	622	–29556.2795	669	–29556.1063
576	–29556.4903	623	–29556.2785	670	–29556.1057
577	–29556.4888	624	–29556.2775	671	–29556.1053
578	–29556.4885	625	–29556.2775	672	–29556.1048
579	–29556.4867	626	–29556.2768	673	–29556.1037
580	–29556.4861	627	–29556.2748	674	–29556.1030
581	–29556.4858	628	–29556.2742	675	–29556.1028
582	–29556.4856	629	–29556.2732	676	–29556.0981
583	–29556.4851	630	–29556.2726	677	–29556.0931
584	–29556.4851	631	–29556.2709	678	–29556.0145
585	–29556.4850	632	–29556.2702	679	–29556.0129
586	–29556.4848	633	–29556.2701	680	–29556.0079
587	–29556.4834	634	–29556.2698	681	–29556.0055
588	–29556.4831	635	–29556.2683	682	–29556.0043
589	–29556.4792	636	–29556.2679	683	–29555.9979
590	–29556.4172	637	–29556.2657	684	–29555.9959
591	–29556.4134	638	–29556.2643	685	–29555.9932
592	–29556.4042	639	–29556.2624	686	–29555.9899
593	–29556.3920	640	–29556.2591	687	–29555.8784
594	–29556.3872	641	–29556.2555	688	–29555.8657
595	–29556.3852	642	–29556.2527	689	–29555.7831
596	–29556.3839	643	–29556.2495	690	–29555.7627
597	–29556.3788	644	–29556.2444	691	–29555.7576
598	–29556.3787	645	–29556.2427	692	–29555.7454

693	–29555.7439
694	–29555.7393
695	–29555.7288
696	–29555.7238
697	–29555.7219
698	–29555.7204
699	–29555.7196
700	–29555.7183
701	–29555.7173
702	–29555.7125
703	–29555.7121
704	–29555.7120
705	–29555.7114
706	–29555.7085
707	–29555.7062
708	–29555.7053
709	–29555.7032
710	–29555.7032
711	–29555.7031
712	–29555.7016
713	–29555.7005
714	–29555.7003
715	–29555.7003
716	–29555.7002
717	–29555.6964
718	–29555.6963
719	–29555.6937
720	–29555.6921
721	–29555.6917
722	–29555.6916
723	–29555.6898
724	–29555.6874
725	–29555.6873
726	–29555.6865
727	–29555.6859
728	–29555.6857
729	–29555.6850
730	–29555.6848
731	–29555.6848
732	–29555.6844
733	–29555.6827
734	–29555.6738
735	–29555.6601
736	–29555.6545
737	–29555.6491
738	–29555.6463
739	–29555.6441
740	–29555.5964
741	–29555.5934
742	–29555.5931
743	–29555.5886
744	–29555.5884
745	–29555.5879
746	–29555.5834
747	–29555.5818
748	–29555.5818
749	–29555.5800
750	–29555.5793
751	–29555.5769
752	–29555.5751
753	–29555.5751
754	–29555.5714
755	–29555.5689
756	–29555.5651
757	–29555.5629
758	–29555.5625
759	–29555.5597
760	–29555.5474
761	–29555.5414
762	–29555.4413
763	–29555.1780
764	–29555.1679
765	–29555.1590
766	–29555.1568
767	–29555.1316
768	–29555.1259
769	–29555.1194
770	–29555.1094
771	–29555.1093
772	–29555.0969
773	–29555.0880
774	–29554.9536
775	–29554.9409
776	–29554.9361
777	–29554.9325
778	–29554.9223
779	–29554.9196
780	–29554.9195
781	–29554.9122
782	–29554.9122
783	–29554.9107
784	–29554.9084
785	–29554.9076
786	–29554.9051
787	–29554.9047
788	–29554.9021
789	–29554.9019
790	–29554.9011
791	–29554.9010
792	–29554.9005
793	–29554.8957
794	–29554.8923
795	–29554.8900
796	–29554.8256
797	–29554.7112
798	–29554.7087
799	–29554.7085
800	–29554.7047
801	–29554.6438
802	–29554.6403
803	–29554.6230
804	–29554.6189
805	–29554.6188
806	–29554.6139
807	–29554.5261
808	–29554.5168
809	–29554.3761
810	–29554.3729
811	–29554.3674
812	–29554.3631
813	–29554.1147
814	–29553.8417
815	–29553.8128
816	–29553.5188
817	–29553.5185
818	–29553.4803
819	–29553.3968

Table S6.2: Total enthalpies of fully-optimised structures (see Figure 2).

Index	Enthalpy / eV
1	–31019.110225
2	–31019.110155
3	–31019.110058
4	–31019.110055
5	–31019.109645
6	–31019.109543
7	–31019.109377
8	–31019.109367
9	–31019.109335
10	–31019.109328
11	–31019.109311
12	–31019.109287
13	–31019.109261
14	–31019.109241
15	–31019.109155
16	–31019.109141
17	–31019.109018
18	–31019.109010
19	–31019.108995
20	–31019.108938
21	–31019.108838
22	–31019.108820
23	–31019.108709
24	–31019.108670
25	–31019.108666
26	–31019.108631
27	–31019.108590
28	–31019.108573
29	–31019.108221
30	–31019.108031
31	–31019.107959
32	–31019.107807
33	–31019.107431
34	–31019.107088
35	–31019.106923
36	–31019.106895
37	–31018.671057
38	–31018.670534
39	–31018.670421
40	–31018.670184
41	–31018.670184
42	–31018.670125
43	–31018.670039
44	–31018.670010
45	–31018.669925
46	–31018.669913
47	–31018.669885
48	–31018.669885
49	–31018.669837
50	–31018.669804
51	–31018.669799
52	–31018.669789
53	–31018.669727
54	–31018.669655
55	–31018.669471
56	–31018.669405
57	–31018.669280
58	–31018.669040
59	–31018.669029
60	–31018.668788
61	–31018.668482
62	–31018.667846
63	–31018.667805
64	–31018.585085
65	–31018.584103
66	–31018.584036
67	–31018.537492
68	–31018.536166
69	–31018.534626
70	–31018.533708
71	–31018.531184
72	–31018.504152
73	–31018.503743
74	–31018.502555
75	–31018.501984
76	–31018.451537
77	–31018.358249
78	–31018.355731
79	–31018.355494
80	–31018.352805
81	–31018.253577
82	–31018.137916
83	–31018.004477
84	–31017.980107
85	–31017.888192
86	–31017.879992
87	–31017.879901
88	–31017.675581
89	–31017.431967
90	–31017.336042
91	–31017.292564
92	–31017.286389
93	–31017.183684
94	–31017.042073
95	–31016.974390
96	–31016.729304
97	–31016.727409
98	–31016.342548
99	–31016.236552
100	–31016.027601
101	–31015.789085
102	–31015.618683
103	–31015.462839
